# Rigid and electrode-compatible multicomponent organic crystals for piezoelectric energy harvesting

**DOI:** 10.1039/d6ta01463a

**Published:** 2026-04-30

**Authors:** Suman Bhattacharya, Maria Zubair, Pierre-André Cazade, Jonathan Moffat, Tara E. Ryan, Krishna Hari, Sarah Guerin

**Affiliations:** a Department of Chemical Sciences, Bernal Institute, University of Limerick Limerick V94 T9PX Ireland Sarah.Guerin@ul.ie; b Department of Chemistry, University of Maynooth Co. Kildare Ireland; c Park Systems UK Ltd Medicity Nottingham D6 Building, Thane Road Nottingham NG90 6BH UK; d SSPC, The Research Ireland Centre for Pharmaceuticals, University of Limerick Limerick V94 T9PX Ireland

## Abstract

Organic piezoelectric materials are entering a new era of discovery and design, where properties can be engineered at the nanoscale using crystal engineering principles. As well as single-component piezoelectric molecular crystals, materials scientists can use multicomponent materials such as cocrystals and metal–organic frameworks to develop tailored, sustainable sensing and actuating materials. Here, we present a multicomponent crystal (MCC), l-argininium amidosulfonate hemihydrate (Sa·l-Arg·0.5H_2_O), which exhibits a maximum local longitudinal piezoelectric response of *d*_33_ = 3.53 pC N^−1^, matching the ideal predicted longitudinal piezoelectric response of *d*_22_ = 3.11 pC N^−1^, and predicted shear piezoelectric response of *d*_36_ = 4.47 pC N^−1^. Sa·l-Arg·0.5H_2_O is scaled up, and self-assembled into stand-alone polycrystalline discs. The polycrystalline discs were successfully electroded with diverse methods, including Cu tapes, Al tapes, and Ag nanoparticles deposited on Carbon cloth (Ag@CC). The open-circuit voltage of the electroded disc of Sa·l-Arg·0.5H_2_O revealed a maximum voltage of 14.5 V for the Cu, and 14.2 V for the Al electrodes, respectively, when subjected to manual tapping, simulating a common mechanical action. Sa·l-Arg·0.5H_2_O represents the first multicomponent piezoelectric crystal disc in our research that simultaneously demonstrates high voltage output, low surface roughness, and high mechanical strength without a corresponding increase in brittleness.

## Abstract 2

Piezoelectric l-argininium amidosulfonate hemihydrate, shapeable into polycrystalline discs electroded with flexible Cu and Al tapes, exhibited an output voltage of **15** V in response to mechanical stimuli in the form of finger tapping.

## Graphical abstract

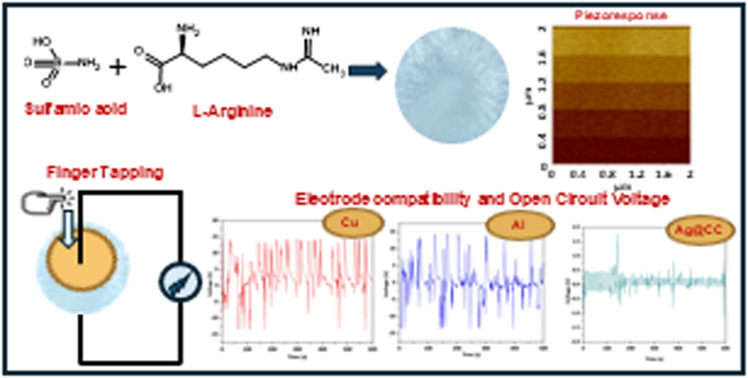

## Introduction

Piezoelectricity, discovered in 1880 by Jacques Curie and Pierre Curie in Rochelle salt,^1^ enables the linear interconversion of electrical and mechanical energy, making it a sustainable energy resource. A 3^rd^ rank tensor, piezoelectricity is an anisotropic and symmetry-dependent property.^2^ For a material to be piezoelectric, a non-centrosymmetric lattice is a primary requisite. Out of 32 crystal classes, 21 classes do not have a center of symmetry, and materials belonging to 20 of them exhibit piezoelectricity. Piezoelectric materials act as electromechanical transducers, converting electrical signals into mechanical strain, and are widely used in imaging, actuation, and structural health monitors.^3–6^ Traditional piezoelectric materials, *e.g.*, quartz (*d*_11_ = −2.3 pC N^−1^, *d*_14_ = 0.7 pC N^−1^), zinc oxide (*d*_33_ = 5 pC N^−1^), aluminium nitride (*d*_33_ = 8 pC N^−1^), and lead zirconium titanate (*d*_33_ = 350–550 pC N^−1^), remain gold standards for their piezoelectric response, devisability, and mechanical stability. However, these materials suffer from several drawbacks, *e.g.*, limited structure–property control, complex synthetic protocols, and the presence of toxic inorganic constituents. This resulted in a paradigm shift towards exploring organic materials,^7–9^*e.g.*, wood, bones, fibrous proteins, bioactive polymers, and collagen, as potential alternatives to conventional inorganic piezoelectric materials. Organic materials, *e.g.*, *γ*-glycine (*d*_33_ = 9.93 pm V^−1^),^10–12^ and diphenylalanine (*d*_33_ range ≈ 5–50 pC N^−1^)^13–15^ have been studied extensively for their electromechanical properties. Recent studies have employed covalent-coordinate chemistry to synthesise piezoelectric metal–organic materials.^16–20^ However, such materials are often discovered serendipitously and involve a complex synthetic procedure. Multicomponent crystals (MCCs),^21,22^*viz.*, cocrystals, ionic cocrystals, salts, hydrates/solvates, solid solutions, produced by cocrystallisation of a parent molecule with a precisely selected coformer, guided by crystal engineering principles,^23^ are novel materials that offer improved structure–property control. Piezoelectric MCCs and organic crystals are gaining significant interest in the scientific community.^24–28^ Kushwaha *et al.* reported a cocrystal of diisopropylammonium bis(4-nitrophenyl) phosphate (DIPA·BNPP), that upon pellet fabrication, exhibited a maximum macroscale *d*_33_ of 7.9 pC N^−1^, measured *via* the Berlincourt method.^29^ Ji *et al.* reported cocrystals of 4,4′-bipyridine and *N*-acetyl-l-alanine, which recorded a *d*_max_ of 14.9 pC N^−1^, and demonstrated a structure–property relation between a phase transition and observed piezoelectric response.^30^ Recently, Sahoo *et al.* presented an exhaustive study on the ferroelectric ionic cocrystal, (*p*-TEA)(*p*-TEAH)·PF_6_, which recorded a macroscopic longitudinal piezoelectric coefficient (*d*_33_) of 4 pC N^−1^, and a piezoelectric voltage coefficient of 38.41 × 10^−3^ V m N^−1^.^31^ Synthesis of MCCs offers an alternative route to harness the piezoelectric potential of materials, *e.g.*, amino acids, whose chemical nature, zwitterionic character, and limited solubility in organic solvents hinder their utilisation for piezoelectric applications. However, integrating the active piezoelectric layer, *viz.*, films, single crystals, or stand-alone elements, with electrodes remains challenging and is a crucial factor influencing overall electrical efficiency. Prevalent electroding methods like sputtering, thermal evaporation, screen printing, and conductive adhesive tapes each present numerous benefits in terms of cost-effectiveness, flexibility, and simplicity.^32–34^ Piezoelectric elements are grown on flexible aluminium foil,^35–37^ and Indium Tin Oxide (ITO) coated sodalime glass,^38,39^ or polyethylene terephthalate,^40,41^ with and without soldered wires. Nonetheless, for organic piezoelectric materials, *e.g.*, amino acids, electroding becomes more challenging because of their fragility and thermal sensitivity, making them incompatible with traditional high-temperature or vacuum-based electrode deposition processes. Additionally, achieving durable and reliable adhesion between electrodes and such delicate piezoelectric active layers is challenging, often leading to poor electrical contact and reduced lifespan of the device.^42,43^

**Fig. 1:**
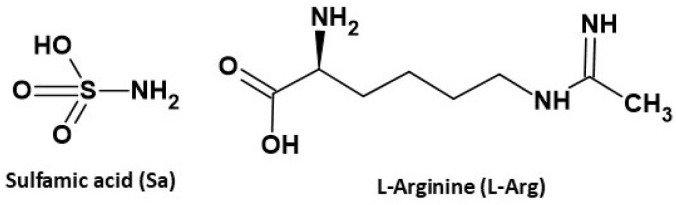
The molecular structure of the coformers forming the hemihydrate salt l-argininium amidosulfonate hemihydrate (Sa·l-Arg·0.5H_2_O).

Herein, we report a salt hydrate, l-argininium amidosulfonate hemihydrate (Sa·l-Arg·0.5H_2_O) derived from l-arginine (l-Arg) and sulfamic acid (Sa) (Fig. 1), which exhibits considerable macroscopic longitudinal piezoelectric response, is satisfactorily scalable, and can be fabricated as stand-alone polycrystalline circular discs. The disc is electroded using diverse conductive materials (*e.g.*, copper, aluminium and Ag deposited on carbon cloth; Ag@CC), due to its low brittleness, high mechanical strength, and low surface roughness, allowing it to be validated for piezoelectric energy harvesting applications.

## Results

### Crystal structure of Sa·l-Arg·0.5H_2_O

**Fig. 2:**
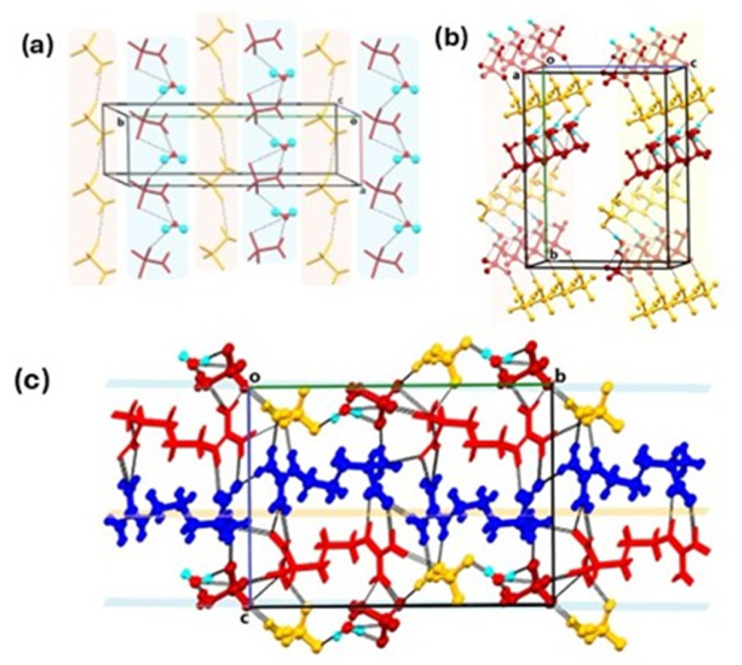
Crystal structure of Sa·l-Arg·0.5H_2_O. Formation of (a) hydrogen bonded chains of symmetrically independent (Sa^−^) (yellow and maroon capped sticks), along the crystallographic *a* axis; (b) corrugated sheets of (Sa^−^) along the *ab* plane; (c) the parallel alignment of the corrugated hydrogen bonded sheets of the symmetrically independent (Sa^−^) and the (l-Arg^+^) (blue ball and sticks), with the other symmetrically independent (l-Arg^+^) (red capped sticks).

Sa·l-Arg·0.5H_2_O crystallises in a monoclinic *P*2_1_ space group with two units of sulfamate anions (Sa^−^) and two units of l-argininium cations (l-Arg^+^) along with a single molecule of H_2_O (Fig. S1 and Table S1) in the asymmetric unit. The protonation of the guanidino moiety of l-Arg occurs by deprotonation of Sa. The three S–O bond distances are respectively 1.454(1) Å, 1.457(1) Å, and 1.446(1) Å, indicating delocalisation. A three-dimensional network of hydrogen bonds (Table S2) sustains the crystal structure of Sa·l-Arg·0.5H_2_O. One set of symmetrically unique (Sa^−^) (yellow capped sticks), forms a hydrogen bonded chain aided by N9–H9C⋯O6, parallel to the crystallographic *a* axis (Fig. 2a). The other set of symmetrically unique (Sa^−^) (maroon capped sticks), forms another hydrogen bonded chain mediated by the water molecule, *viz.*, N10–H10D⋯O11 and O11–H11D⋯O8, parallel to the crystallographic *a* axis (Fig. 2a and b). The parallel running chains of symmetrically independent (Sa^−^) forms a corrugated plane, represented by the blue average plane, along the *ab* crystallographic plane (Fig. 2c), aided by hydrogen bonds O11–H11C⋯O5. Another corrugated sheet formed by symmetrically independent (l-Arg^+^) moieties formed by N7–H7A⋯O3, runs parallel to the corrugated sheets of (Sa^−^). The void between the three corrugated sheets is occupied by columns of another symmetrically independent (l-Arg^+^) moiety (red-capped sticks), linked by hydrogen bonds with surrounding (l-Arg^+^) and (Sa^−^) moieties of the sheets. The bulk purity of Sa·l-Arg·0.5H_2_O was confirmed from the similarity of the calculated powder pattern and experimental powder pattern, as shown in Fig. S2a. The TGA-DSC analyses of Sa·L-Arg·0.5H_2_O is presented in Fig. S2b. It can be seen from the TGA plot that Sa·l-Arg·0.5H_2_O exhibits an onset of weight loss due to the removal of the water of crystallisation at 55 °C. A weight loss of 3.64% is observed up until ≈115 °C which corresponds to 1 molecule of water in the asymmetric unit, and in agreement with the single crystal data. The DSC plot of Sa·l-Arg·0.5H_2_O reveals a broad endotherm corresponding to the loss of water of crystallisation, within the temperature range of 55–115 °C, followed by another sharp endotherm at 135 °C, which corresponds to the decomposition of the material. Attempts to isolate the anhydrous form of Sa·l-Arg·0.5H_2_O and phase determination by PXRD analyses failed, due to decomposition and charring of the treated material. This is the likely reason for the continuous weight loss observed in the TGA plot.

### DFT predicted electromechanical properties of Sa·l-Arg·0.5H_2_O

The DFT predicted single-crystal electromechanical properties of Sa·l-Arg·0.5H_2_O are summarised in Table 1. Sa·l-Arg·0.5H_2_O crystallises in the *P*2_1_ space group, and calculations were carried out to conserve the symmetry of the crystal. The predicted elastic stiffness constants (*C*_*kj*_) are represented as a 6 × 6 matrix as shown in Table 1a. Sa·l-Arg·0.5H_2_O has considerable longitudinal stiffness, *i.e.*, *C*_11_ (35.63 GPa), *C*_22_ (37.04 GPa), and *C*_33_ (53.34 GPa). The shear stiffness constants, *i.e.*, *C*_44_ (9.14 GPa), *C*_55_ (11.62 GPa), and *C*_66_ (8.96 GPa) are comparatively (and expectedly) lower as molecular crystals are prone to forming slip planes that move freely under a shearing force. The considerably higher stiffness constants of Sa·l-Arg·0.5H_2_O are expected for a system whose structure is sustained by strong hydrogen bonds.

DFT predicted (a) elastic stiffness constants [*C*_*kj*_] (GPa); (b) piezoelectric charge constants [*e*_*ij*_] (C m^−2^); (c) piezoelectric strain constants [*d*_*ij*_] (pC N^−1^); (d) piezoelectric voltage tensors [*g*_*ij*_] (mV m N^−1^); (e) dielectric constants

**Table:** 

(a) Elastic stiffness constants [*C*_*kj*_] (GPa)						
*C*_*kj*_	1	2	3	4	5	6
1	**35.63**	15.39	21.514	0.00	0.35	0.00
2	15.39	**37.04**	11.524	0.00	−1.70	0.00
3	21.51	11.52	**53.34**	0.00	2.63	0.00
4	0.00	0.00	0.00	**9.14**	0.00	−0.12
5	0.35	−1.70	2.63	0.00	**11.62**	0.00
6	0.00	0.00	0.00	−0.12	0.00	**8.96**

**Table:** 

(b) Piezoelectric charge constants [*e*_*ij*_] (C m^−2^)						
*e*_*ij*_	1	2	3	4	5	6
1	0.00	0.00	0.00	**0.024**	0.00	**0.054**
2	**0.044**	**0.113**	**0.073**	0.000	**0.016**	0.000
3	0.00	0.00	0.00	**0.024**	0.00	**−0.042**

**Table:** 

(c) Piezoelectric strain constants [*d*_*ij*_] (pC/N)						
*d*_*ij*_	1	2	3	4	5	6
1	0.00	0.00	0.00	**2.752**	0.00	**6.107**
2	**−0.666**	**3.113**	**0.889**	0.00	**1.631**	0.00
3	0.00	0.00	0.00	**2.543**	0.00	**−4.468**

**Table:** 

(d) Piezoelectric voltage tensors [*g*_*ij*_] (mV m N^−1^)						
*g*	1	2	3	4	5	6
1	−0.001	0.004	0.001	**108.06**	0.002	**264.31**
2	**−29.18**	**136.41**	**38.95**	0.009	**71.49**	−0.002
3	−0.001	0.006	0.002	**100.45**	0.003	**−210.12**

**Table:** 

(e) Dielectric constants [*ε*]			
*ε*	1	2	3
1	**2.519**	0.000	0.250
2	0.000	**2.531**	0.000
3	0.250	0.000	**2.854**

The *P*2_1_ space group allows eight non-zero piezoelectric tensor components including longitudinal (*d*_22_), transverse (*d*_21_, *d*_23_), and shear responses. The piezoelectric charge tensor (eighteen components in Voigt notation) of Sa·l-Arg·0.5H_2_O, obtained by DFT calculations, is presented in Table 1b. It is evident that the charge tensors vary from 0.044 C m^−2^ (*e*_21_), 0.024 C m^−2^ (*e*_14_, *e*_34_) to 0.113 C m^−2^ (*e*_23_). Experimentally, the strain constants are more readily accessible, and their predicted values are shown in Table 1c. Amongst the 8 non-zero piezoelectric strain constants, which represent the product of polarisation and mechanical compliance in matrix form, Sa·l-Arg·0.5H_2_O exhibits a predicted longitudinal piezoelectric strain constant of 3.11 pC N^−1^ (*d*_22_), and transverse piezoelectric strain constants of −0.67 pC N^−1^ (*d*_21_) and 0.89 pC N^−1^ (*d*_23_). Further, larger shear constants of −4.47 pC N^−1^ (*d*_36_), 6.11 pC N^−1^ (*d*_16_), 2.75 pC N^−1^ (*d*_14_), 2.54 pC N^−1^ (*d*_34_), and 1.63 pC N^−1^ (*d*_25_) are also predicted. This is due to the weak elastic shear components, which enable larger deformation of the crystal along its angles, resulting in larger polarisations. Though comparable to widely used piezoelectrics such as quartz, zinc oxide, and aluminium nitride, the strain constants are relatively low. However, the low dielectric constants 2.519, 2.531, 2.854 (Table 1e) result in high predicted piezoelectric voltage constants, ranging from 29−264 mV m N^−1^, (Table 1d), compared to an average of 40 and 250 mV m N^−1^ for commercial PZT and PVDF, respectively.^44,45^

### Fabrication of Sa·l-Arg·0.5H_2_O into polycrystalline discs

The scalability of Sa·l-Arg·0.5H_2_O enabled the fabrication of polycrystalline discs with a consistent diameter of 3.5 cm (Fig. S3a) and a thickness ranging from 5.6 to 7.2 mm (Fig. S3b) with considerably smooth surfaces. SEM images of the surface of the polycrystalline discs revealed condensed packing of aligned microneedle-shaped crystallites of Sa·l-Arg·0.5H_2_O (Fig. S4a) spread into domains of smooth surfaces in between rough domains formed by ordered microneedles protruding out of the surface. The SEM images of the cross-section of the discs (Fig. S4b) confirmed the compact directional packing of the crystallites. The aligned crystallites indicate that more directional dipoles are oriented along the applied mechanical stress, and the dense packing and smooth upper and inverted surfaces ensure stable electrical contact with the electrodes, thereby facilitating efficient mechanical-to-electrical energy conversion.

The polycrystalline circular discs were subjected to compression testing to assess their durability under applied pressure. A set of four polycrystalline discs of Sa·l-Arg·0.5H_2_O of 3.5 cm diameter and 4.72 mm average thickness placed rough side down were subjected to a uniaxial force from 0–5% strain by a presser. The pressure was then lifted, and the process was repeated three times at the same location. The initial linear region of the stress–strain plot was used to determine the apparent Young's moduli (Fig. S4c), yielding values of 132.9, 106.9, 39.5, and 18.3 GPa, corresponding to yield-strength force levels of 7, 9, 14, and 105 N, respectively. The experimentally observed Young's moduli of the polycrystalline discs of Sa·l-Arg·0.5H_2_O confirm their rigidity and facile handleability. The mean value exceeds the majority of molecular crystal piezoelectrics reported to date, approaching the “metal-like rigidity” of diphenylalanine-derivative peptide assemblies.^46^ The discs were found to form multiple cracks before total disintegration occurred. The polycrystalline nature of the sample results in a large distribution of measured values at the macroscale, and even in the stress–strain intercepts, as the initial fracture from which the Young's Modulus is estimated will occur to differing extents and along different planes each time.

### Macroscopic and nanoscale piezoelectric response of Sa·l-Arg·0.5H_2_O

**Fig. 3:**
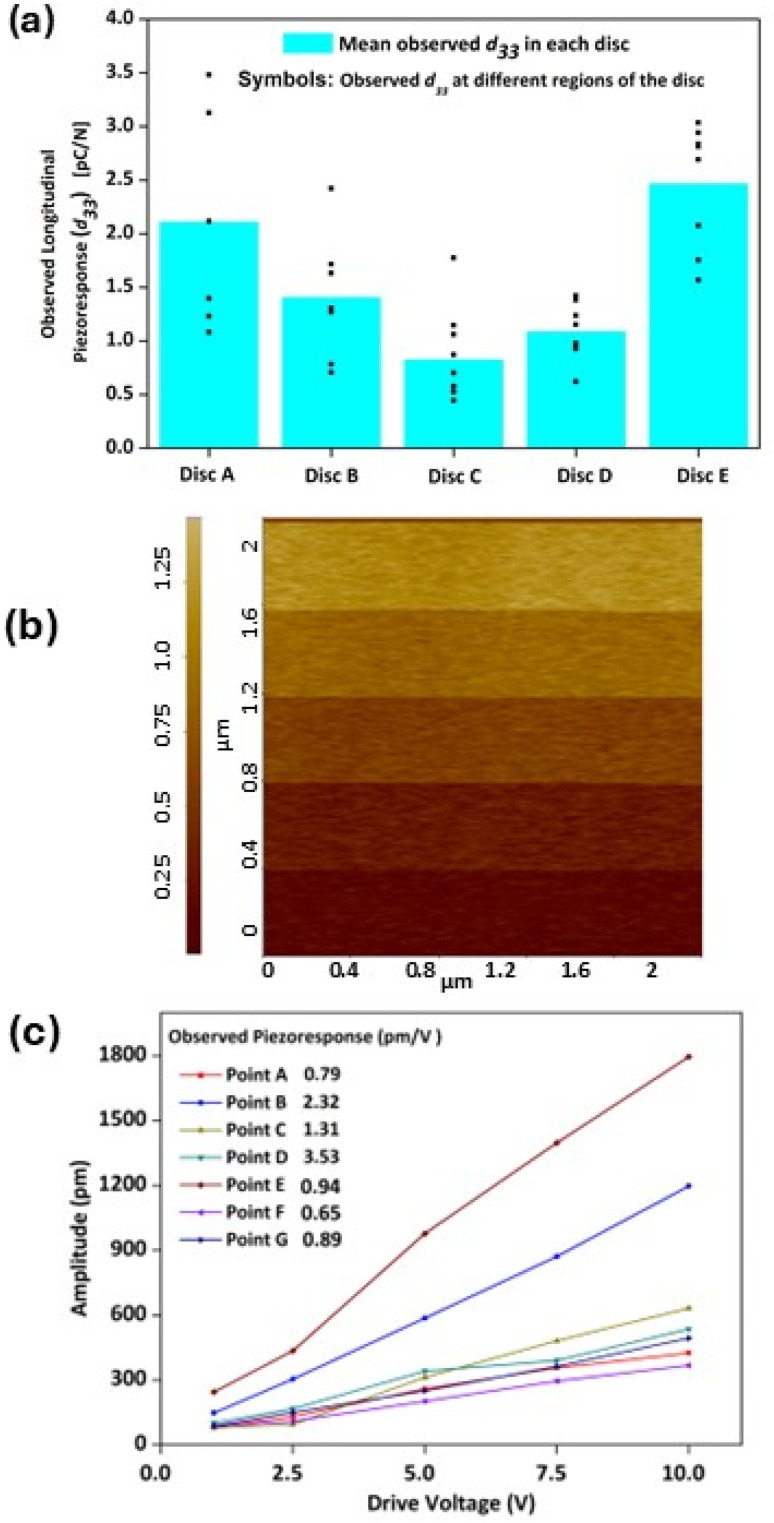
(a) Average longitudinal piezoelectric response (*d*_33_) observed in discs of Sa·l-Arg·0.5H_2_O (shown as turquoise bars) and the range of effective *d*_33_ observed in different positions in a disc (shown in symbols); (b) representative example of the response amplitude of a PFM scan showing a staircase effect of response amplitude with increase in the driving voltage; (c) the quantified longitudinal piezoelectric response obtained in PFM measurements.

The macroscopic piezoelectric response of Sa·l-Arg·0.5H_2_O was assessed using five polycrystalline discs, with eight sets of measurements recorded at different regions across each disc. The average effective longitudinal (*d*_33_) response of Sa·l-Arg·0.5H_2_O was found to range from 0.82–2.48 pC N^−1^ across the five discs (Fig. 3a). The effective mean *d*_33_ response (pC N^−1^) within a single disc, *e.g.*, disc A, varied between 1.08–3.48, and for disc C it varied 0.45–1.15. A single crystal longitudinal piezoelectric response (*d*_33_) is crystallographically restricted for a material crystallised in the *P*2_1_ space group (Table 1). However, across five polycrystalline discs of Sa·l-Arg·0.5H_2_O, an effective macroscopic piezoelectric response ranging between 0.82–2.48 pC N^−1^ is observed and is presented in Fig. 3a. As the surface of the polycrystalline discs represents a conglomeration of randomly oriented crystal faces, these local variations occur due to the crystal faces that get exposed to the electrodes of the piezometer. Such an occurrence of effective macroscopic longitudinal piezoelectric response (*d*_33_) was also observed in l-lysinium *S*-(+)-mandelate pentahydrate, which recorded an effective macroscopic *d*_33_ of 11 pC N^−1^, exceeding a theoretical prediction of 3.5 pC N^−1^, attributable to shear piezoelectric responses such as *d*_36_ (10.8 pC N^−1^).^27^ The contribution from diverse crystal faces to the experimentally measured *d*_33_ was confirmed from Piezoresponse Force Microscopy (PFM) measurements carried out on randomly selected local regions of Sa·l-Arg·0.5H_2_O. To determine the nanoscale piezoelectric coefficient, the driving amplitude of the AC signal increased in steps: 1 V, 2.5 V, 5 V, 7.5 V, and 10 V whilst scanning over an area of 2 × 2 µm. A staircase effect is observed in the amplitude signal, with the response amplitude increasing with increasing driving amplitude (Fig. 3b). Quantification of the PFM responses revealed a near-identical varying piezoelectric response ranging between 0.65–3.53 pm V^−1^ (Fig. 3c), which were consistent with the observed macroscopic piezoelectric responses of Sa·l-Arg·0.5H_2_O. The PFM Topography, Phase, and Amplitude maps are presented in Fig. S5. The material Sa·l-Arg·0.5H_2_O crystallises in the non-centrosymmetric *P*2_1_ space group, suggesting that the material may exhibit ferroelectric properties. Sa·l-Arg·0.5H_2_O discs were tested for ferroelectricity, but no switching or hysteresis was observed at the nanoscale (bias of 10 V using PFM), or the macroscale (bias of 100 V using a commercial ferroelectric tester). Switching may be obtainable at higher driving voltages beyond the capabilities of our test equipment.

### Piezoelectric energy harvesting with Sa·l-Arg·0.5H_2_O

To investigate the electrode compatibility of Sa·l-Arg·0.5H_2_O with different electrodes and its performance as piezoelectric energy harvester, three polycrystalline discs were prepared and individually electroded using: (i) flexible copper (Cu) tape, (ii) flexible aluminium (Al) tape, both offering reliable electrical contact and mechanical stability, and (iii) Ag@CC, which provided a high surface area and enhanced charge collection due to the presence of uniformly distributed conductive silver nanoparticles. This was ensured during electroding of Sa·l-Arg·0.5H_2_O discs, so that the electrodes (Cu, Al, Ag@CC) cover the total surface of the discs. Even though local variations were observed in macroscopic piezoelectric response of the discs (Fig. 3a), the charge generated due to the application of mechanical stimuli represents the overall charge generation across the surface of the disc, and not a local effect. Thus, the varying macroscopic piezoelectric response of the discs will expectedly not impact the energy harvesting performance of the discs. Aluminium and copper tapes were purchased and employed without any surface treatment, while Ag@CC was prepared by following the procedures elaborated in the Experimental section and Fig. S6a. The simple, cost-effective solution-based preparation of Ag@CC ensured uniform deposition of silver nanoparticles onto the complex 3D surface of the carbon cloth (CC), and precise control over particle size and loading by tuning reaction conditions, without high-temperature processing. Additionally, the wet-chemical method ensures good interaction between the nanoparticles and the carbon substrate, which is crucial for achieving stable and efficient electrode performance. Morphological and structural analysis of free-standing Ag@CC electrodes were carried out using SEM and PXRD (Fig. S6b and S6c). The SEM images of bare CC and Ag@CC reveal that CC is well covered with the Ag nanoparticles. The average size of the particles was 100 nm. The elemental composition analysis of Ag@CC was carried out using the SEM-EDX analysis (Fig. S7a) which reveals the presence of carbon 60% and Ag (19.26%), confirming the growth of silver nanoparticles on CC fibres. The PXRD patterns of the bare CC show a broad peak at 25.8°, while for Ag@CC, additional peaks at 38.3° and 44.5° are observed, which are attributed to the deposition of Ag in Ag@CC (Fig. S6c). The prominent peak at 38.3° can be attributed to the (111) plane of the cubic phase of silver.^47,48^ The Raman spectra (Fig. S7b) of Ag@CC show peaks around 1350 cm^−1^ and 1580 cm^−1^, corresponding to D and G bands of the CC, respectively.^49,50^ The appearance of a small peak at around 995 cm^−1^ is attributed to deposited silver nanoparticles.

**Fig. 4:**
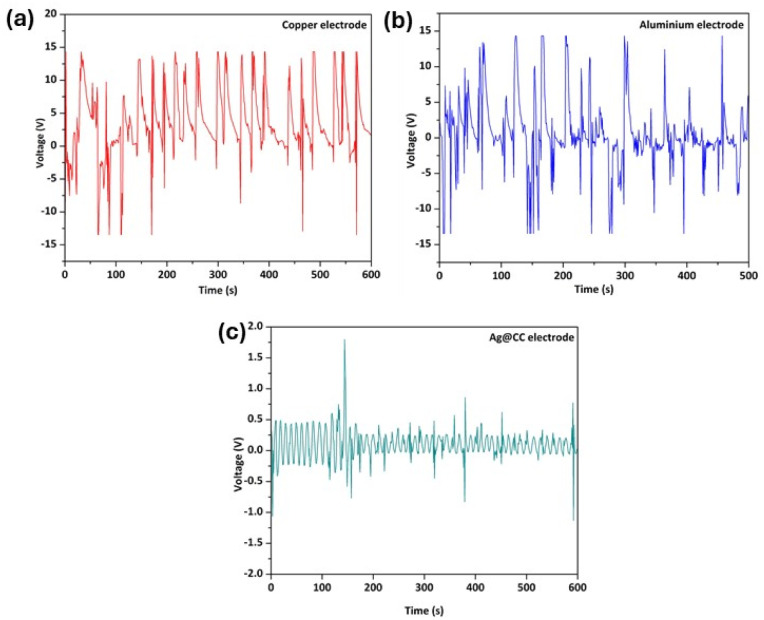
The OCVs observed for discs of Sa·l-Arg·0.5H_2_O when electroded with (a) flexible Cu tape; (b) flexible Al tape; and (c) Ag@CC.

Fig. 4 shows the open-circuit voltage (OCV) generated by polycrystalline discs of Sa·l-Arg·0.5H_2_O. To simulate a real-world mechanical stimulus, the electroded discs of Sa·l-Arg·0.5H_2_O were tapped manually with varying intensities using the human finger, yielding varying shapes and intensities of the voltage peaks. Depending on the electrode material used, the polycrystalline assemblies demonstrated remarkable sensitivity, producing maximum peak-to-peak voltages in the range of 1.79 to 15 V even with gentle tapping. A maximum output voltage of ≈14.5 V and ≈14.2 V were obtained for the Cu and Al electroded discs, respectively (Fig. 4a and b). The maximum peak-to-peak voltages for Cu and Al electroded discs, respectively, were recorded to be 26.1 V and 24.8 V. However, in the case of the Ag@CC electroded disc, the maximum recorded output voltage and peak-to-peak voltage were recorded to be ≈1.79 V and 2.37 V, respectively (Fig. 4c). The higher output voltage from flexible Cu and Al electrodes can be attributed to their higher electrical conductivity and greater material stability, as well as more efficient adhesion with the piezoelectric active layer of the disc, compared to Ag@CC.

## Conclusions

Sa·l-Arg·0.5H_2_O, a salt hydrate, has been engineered with repeatedly low surface roughness, high rigidity, low brittleness, and high voltage output across any number of samples produced. Sa·l-Arg·0.5H_2_O exhibited a macroscopic longitudinal piezoelectric response of 0.82–2.48 pC N^−1^ across multiple samples, attributable to contributions from different crystal faces present on the disc surfaces. For the first time in organic piezoelectrics, the energy-harvesting performance of the polycrystalline discs of Sa·l-Arg·0.5H_2_O was benchmarked across three physicochemically distinct electrodes, including Al and Cu tapes and a new Ag@CC. This work demonstrates that scalable MCCs with consistent piezoelectric response can be fabricated into standalone polycrystalline discs, highlighting their versatility and potential for use as piezoelectric energy harvesters. By overcoming the fragility and surface roughness of previous organic polycrystalline piezoelectrics, this study aims to accelerate the commercial uptake of these sustainable piezoelectrics, most predominantly as high-performance energy harvesting components.

## Author contributions

S. B.: synthesis and characterisation, validation, methodology, investigation, formal analysis, data curation, conceptualization, manuscript preparation; M. Z.: electroding of the discs and energy harvesting experiments; P.-A. C.: DFT calculations; J. M.: PFM measurements, T. R.: compressibility experiments; K. H.: SEM images; S. G.: writing, review & editing, validation, supervision, software, resources, project administration, investigation, funding acquisition, formal analysis, conceptualisation.

## Conflicts of interest

There are no conflicts to declare.

## Supplementary Material

TA-014-D6TA01463A-s001

TA-014-D6TA01463A-s002

## Data Availability

CCDC 2425949 (Sa·l-Arg·0.5H_2_O) contains the supplementary crystallographic data for this paper.^51^ The supporting data has been provided as part of the supplementary information (SI). Supplementary information: experimental and methodology; ORTEP of Sa·L-Arg·0.5H_2_O; crystallographic information table; details of hydrogen bond interaction geometry; PXRD, TG-DSC, SEM, PFM (topography, amplitude, phase) of Sa·L-Arg·0.5H_2_O discs; synthetic details of Ag@CC; SEM, Raman and PXRD of Ag@CC. See DOI: https://doi.org/10.1039/d6ta01463a.
