# Supplementary material for: Rigid and electrode-compatible multicomponent organic crystals for piezoelectric energy harvesting
Source: J Mater Chem A Mater. 2026 Apr 30;14(37):24441–7. doi: 10.1039/d6ta01463a (PMC13148129; doi:10.1039/d6ta01463a)
Supplement: TA-014-D6TA01463A-s001 [file TA-014-D6TA01463A-s001.pdf]

Supplementary Information (SI) for Journal of Materials Chemistry A.
This journal is © The Royal Society of Chemistry 2026

Supplementary Information

Rigid and electrode-compatible multicomponent organic crystals for piezoelectric energy harvesting

Suman Bhattacharya†[1], Maria Zubair†[1], Pierre–Andre Cazade[2], Jonathan Moffat[3], Tara E. Ryan[1], Krishna Hari[1], Sarah Guerin*[1,4]

[1]Department of Chemical Sciences, Bernal Institute, University of Limerick, Limerick V94 T9PX, Ireland

[2]Department of Chemistry, University of Maynooth, Co. Kildare, Ireland

[3]Park Systems UK Ltd., Medicity Nottingham D6 Building, Thane Road, Nottingham, NG90 6BH, United Kingdom

[4]SSPC, The Research Ireland Centre for Pharmaceuticals, University of Limerick, Limerick V94 T9PX, Ireland

Sl. No.	Contents	Page No.
1.	Experimental and Methodology	2
2.	**Figure S1**: ORTEP of **Sa•L-Arg•0.5H$_2$O** at 50% probability	5
	**Table S1**: Crystallographic information table for **Sa•L-Arg•0.5H$_2$O**.	6
3.	**Table S2**: Details of hydrogen bond interaction geometry for **Sa•L-Arg•0.5H$_2$O**.	7
4.	**Figure S2**: (**a**) Comparison showing the similarity of the calculated powder pattern and the experimental powder pattern of **Sa•L-Arg•0.5H$_2$O**, confirming a pure phase bulk synthesis; (**b**) The TG–DSC plot of **Sa•L-Arg•0.5H$_2$O**, revealing a broad endotherm corresponding to the loss of water molecule with an onset ≈ 55 ^{o}C, followed by the decomposition of the anhydrous phase ≈ 150 ^{o}C.	9
5.	**Figure S3**: (**a**) Polycrystalline disc of **Sa•L-Arg•0.5H$_2$O** of diameter = 3.5 cm; (**b**) Average thickness of polycrystalline discs of **Sa•L-Arg•0.5H$_2$O** across five samples.	10
6.	**Figure S4**: (**a**) and (**b**) SEM images of the surface and cross section of the polycrystalline disc of **Sa•L-Arg•0.5H$_2$O** showing condensed packing of aligned micro needle of **Sa•L-Arg•0.5H$_2$O**; (**c**) The elastic region of the Stress-Strain curve, from the initial linear region, of the circular discs obtained under a uniaxial force set to a maximum of 200 N.	
7.	**Figure S5**: PFM topography, PFM Amplitude and PFM phase observed in nanoscale piezoresponse assessment of **Sa•L-Arg•0.5H$_2$O**.	11
8.	**Figure S6**: **a**) Schematic presentation of the steps followed for the Synthesis of **Ag@CC**; **b**) SEM images of (i) bare **CC** (ii) **Ag@CC** and (iii) magnified area for Ag nanoparticles on the **CC**; **c**) PXRD of bare CC and **Ag@CC**.	
9.	**Figure S7** (**a**) SEM EDX results of **Ag@CC**; (**b**) Raman spectra of **Ag@CC**.	12

Experimental and Methodology

Synthesis

All chemicals including L-Arginine, Sulfamic acid, D-(+)-Glucose, Silver nitrate, and carbon cloth used in this work were purchased from Thermofischer Scientific and used without any further purification.

Synthesis of L-Argininium amidosulfonate hemihydrate (Sa•L-Arg•0.5H_2O): Sulfamic acid (**Sa**) (200 mg, 2.06 mmol) and L-arginine (**L-Arg**) (359 mg, 1 eq.) are collected in a mortar and ground with a pestle to achieve a uniform mixture. The 1:1 mixture of the two constituents is then dissolved in 10 mL of distilled water with constant stirring and mild heating ~ 40 – 45 ^{0}C. The clear solution is kept undisturbed for slow evaporation of the solvent. After evaporation of the solvent, a polycrystalline growth appeared, which was manually disintegrated to isolate diffraction quality single crystals, which were characterised by single-crystal X-ray diffraction to be a hemihydrate of the salt, L-Argininium amidosulfonate (**Sa•L-Arg•0.5H_2O**). To make a stand-alone polycrystalline circular disc of 1, a 1:1 mixture of **Sa** (10 g, 0.103 mmol) and **L-Arg** (18 g, 1 eq.), was dissolved in minimum amount of distilled water ~ 25 mL. The solution was drop-casted into flexible silicone circular molds. Slow evaporation of distilled water resulted in the formation of a circular polycrystalline disc of **1**. The thickness of the disc could be varied by putting subsequent crystallisations on the same mold.

Synthesis of Silver @ Carbon Cloth (Ag@CC): The synthesis of silver nanoparticles on carbon cloth (**CC**) was carried out using simple two step methods as has been schematically shown in **Figure S6a**. Firstly, two separate solutions were prepared in distilled water using 0.08 mmol of $AgNO_3$ and 0.05 mmol of D-(+)-Glucose, respectively. First step involved dipping the CC in $AgNO_3$ solution followed by drying on hotplate at 80 ^{o}C for 5 min. Then in the second step Ag ion coated on CC was reduced chemically by dipping in the solution of D-(+)-Glucose. These steps were repeated twenty times to get well coverage of Ag on CC. Excess Ag ions were removed by washing the Ag deposited CC with water and drying at 120 ^{o}C prior to structural and morphological analysis.

Electroding of the polycrystalline disc of Sa•L-Arg•0.5H_2O: To assess the piezoelectric power generation of **Sa•L-Arg•0.5H_2O** disc, we fabricated three coin-sized devices using flexible Aluminium tape (Al), flexible Copper tape (Cu), and **Ag@CC** as an electrode. The disc of **Sa•L-Arg•0.5H_2O** was placed between these electrodes (Al, Cu and **Ag@CC**) to design sandwich devices. Two copper solder wires were connected to the electrodes. Human finger tapping was used as the mechanical stimulus. Open circuit voltage (OCV) measurements were performed using a Bio-Logic system.

Single-crystal x-ray diffraction (SC-XRD): SC-XRD experiments were performed on a Bruker D8 Quest Single Crystal diffractometer, equipped with Mo-Kα radiation source (λ = 0.71073 Å) and Phonon 100 detector. The data collection, integration, and scaling were performed using SMART and SAINT programs, incorporated in APEX 5. The absorption correction was conducted by a multi-scan method using SADABS. Space group determination was performed by XPREP 3 implemented in APEX 5. The structure solution was performed by direct methods

using SHELX[1] and refinement was performed by SHELXL[2] contained in Olex 2 package[3]. All non-hydrogen atoms were refined anisotropically, and all carbon-bonded hydrogens were placed in calculated positions. The temperature was controlled by a liquid nitrogen stream maintained at 150 K using Oxford Cryostream. ORTEP at 50% and crystallographic data for the structure are provided in **Figure S1** and **Table S1**. CIF file containing complete information on **Sa•L-Arg•0.5H$_2$O**, was deposited to CCDC, deposition number **2425949**, and is freely available upon request from the following web site: www.ccdc.cam.ac.Uk/data_request/cif.

Powder X-ray diffraction (PXRD): Powder diffraction experiments were conducted on the polycrystalline disc of **Sa•L-Arg•0.5H$_2$O**, powdered, and ground with a mortar pestle, mounted on zero background disc on a Panalytical Empyrean diffractometer (40 kV, 40 mA, Cu Kα1,2 (λ = 1.5418 Å). A scan speed of 0.111747° /s (6.7° /min), with a step size of 0.02° in 2θ was used at room temperature with a range of 5° < 2θ < 40°. The experimental pattern and the calculated pattern obtained from the single crystal data were matched to ascertain the phase purity.

DFT calculations

All modelling was performed using the CP2K package[4] for periodic density functional theory calculations (DFT)[5]. The Orbital Transformation[6] (OT) SCF algorithm was used with Goedecker, Teter and Hutter (GTH) type pseudopotentials and a molecular optimised double-zeta gaussian basis set[7] (akin to 6–31G**). The cut-off for the plane waves and gaussians was 900 Ry and 60 Ry, respectively, and energy was converged to 10^{-8} hartree. Exchange-correlation effects were treated using the Perdew, Burke, and Ernzerhof (PBE)[8] implementation of the Generalised Gradient Approximation (GGA)[9]. Grimme D3[10,11] van der Waals corrections are also used. All crystal structures were optimised using the Limited-memory Broyden-Fletcher-Goldfarb-Shanno (L-BFGS) algorithm[12] with a supercell model: $4 \times 2 \times 2$ for **Sa-L-Arg**. The piezoelectric and elastic tensors were calculated using a finite differences method, with the supercell strained in each of the Voigt directions ± 0.015. The piezoelectric tensor is then the response of polarization, taken here as a periodically corrected Berry phase, to the applied strain, and the elastic tensor as the response of the stress tensor. The matrix product between the piezoelectric charge tensor, **e**, and the inverse of the elastic tensor (compliance tensor $\mathbf{S}=\mathbf{C}^{-1}$) gives the piezoelectric strain tensor **d**.

Piezoelectric response measurements: Piezoelectric measurements were carried out using a commercial d_{33} piezometer with a numerical accuracy of 0.01 pC/N. The measurement setup is based on the Berlincourt method, with the polycrystalline discs placed between two electrodes, securely clamped and applied with a dynamic force of 2 – 2.5 N at 110 Hz. Further, the crystalline assembly was inverted along the chosen axis to measure the opposite polarity and confirm a genuine longitudinal response. Lead Zirconium Titanate (351 pC/N) references were used for calibration. For a single circular disc, 8 sets of measurements (upright and inverted) were taken, and then the average piezoelectric response was calculated for the disc.

Piezoresponse Force Microscopy

Piezoresponse force microscopy (PFM) was carried out on an NX20 atomic force microscope from Park Systems (Suwon, South Korea) equipped with an ElectriMulti75-G probe from Budget Sensors (Sofia, Bulgaria). PFM measurements were carried out with the probe in contact with the surface whilst an electrical alternating current (AC) is applied to the probe. The amplitude of the probe in response to the piezo effect of the sample was monitored. To determine the piezoresponse coefficient, the driving amplitude of the AC signal was increased in steps: 1V, 2.5V, 5V, 7.5V and 10V whilst scanning an area of 2x2 μm. Scans were performed at a scan rate of 0.5 Hz line rate and at a pixel resolution of 256x256. A staircase effect can be seen in the amplitude signal, representing an increase in the response amplitude with respect to an increase in the driving amplitude. An average response amplitude was then determined for each step and then plotted against the driving voltage. The relationship between response amplitude and driving amplitude was linear, and the piezoresponse coefficient was taken to be the slope of the fitted line.

Compression Testing: A Mecmesin Multi-test 2.5 dv (Mecmesin, West Sussex, UK) device was used for compression testing to compare the strength of the discs at the macroscale. A sample presser of diameter = 2 cm was used, and the sample height was set to an average of 4.72 mm. The maximum force was varied for each disc and were, respectively, 7N, 9N, 14N, and 105N for the four tests. The strain was varied from 0 – 5 % during the tests. The initial linear region of this was plotted and the apparent Young's modulus was calculated from the slope. The maximum strain the sample could withstand before completely breaking was 30%. The samples were placed rough side down in a plastic petri dish that allows bending of samples without interfering with the values, and the loader was applied uniaxially downwards at a steady rate of 1mm/min, which corresponded to 450 data points for 0-1% strain.

Scanning electron microscopy (SEM): Scanning electron microscopy image was used to understand the surface of the polycrystalline discs, revealing the different orientations and packing of the polycrystalline structures. SEM images were taken on a Hitachi SU-70 where the crystals were coated in a 5 nm gold film prior to imaging, using a K550 Emitech Sputter Coater, with a coating current of 20 mA for 90 s. Sputtering was carried out to prevent the organic samples from getting damaged due to high energy electron beam.

Thermogravimetric Analysis: Thermogravimetric analyses (TGA) for **Sa•L-Arg•0.5H_2O** were carried out in Perkin Elmer TGA 4000, between 30 °C – 220 °C, with a constant heating rate of 10 ºC/min.

Differential Scanning Calorimetry: Differential Scanning Calorimetry (DSC) of **Sa•L-Arg•0.5H_2O** was carried out in a Netzsch Polyma 214 between 30 °C – 220 °C, with a constant heating rate of 10 ºC/min.

Figure S1: ORTEP of **Sa•L-Arg•0.5H$_2$O** at 50% probability. Only hydrogen atoms involved in hydrogen bonds and hydrogen atoms bonded to the chiral centres are shown for clarity.

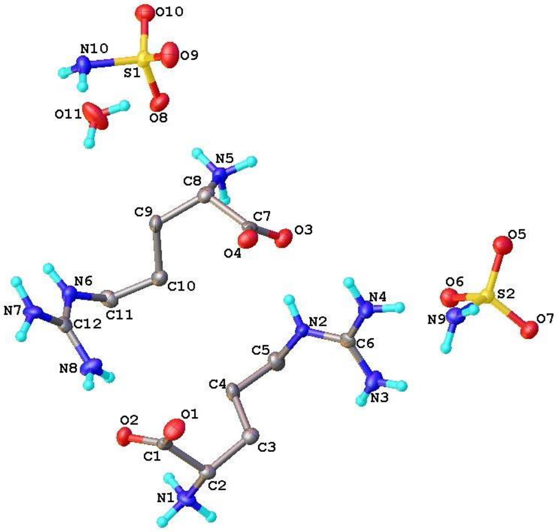

Table S1: Crystallographic information table for **Sa•L-Arg•0.5H_2O**.

Identification code	**Sa•L-Arg•0.5H_2O**
Empirical formula	$C_{12}H_{36}N_{10}O_{11}S_2$
Formula weight/amu	560.63
Temperature/K	150(2)
Crystal system	Monoclinic
Space group	$P2_1$
a/Å	5.2736(2)
b/Å	17.7357(7)
c/Å	13.0228(5)
α/°	90.00
β/°	99.6760(10)
γ/°	90.00
Volume/Å^3	1200.71(8)
Z	2
ρ_{calc}g/cm^3	1.551
μ/mm^{-1}	0.296
F(000)	596.0
Crystal size/mm^3	0.28 × 0.14 × 0.12
Radiation	MoKα (λ = 0.71073)
2Θ range for data collection/°	6.34 to 51.46
Index ranges	-6 ≤ h ≤ 6
	-21 ≤ k ≤ 0
	-15 ≤ l ≤ 15
Reflections collected	4490
Independent reflections	2364<br>R_{int} = 0.0634<br>R_{sigma} = 0.0689
Data/restraints/parameters	2364 / 23 / 399
Goodness-of-fit on F^2	1.103
Final R indexes [I>=2σ (I)]	R_1 = 0.0328
	wR_2 = 0.0703
Final R indexes [all data]	R_1 = 0.0454
	wR_2 = 0.0727
Largest diff. peak/hole / e Å^{-3}	0.25/-0.31
Flack parameter	0.14(14)
**CCDC No.**	**2425949**

a, b, c (Å): Unit cell lengths
α, β, γ (°): Unit cell angles
Z : No. of the formula units in the unit cell.
ρ_{calc} (g/cm^3): Density values calculated from the crystal cell and contents.

μ (mm^{-1}):The absorption coefficient μ in reciprocal millimetres calculated from the atomic content of the cell, the density and the radiation wavelength.

F(000) : The expression for a structure factor evaluated in the zeroth-order case h = k = l = 0, F(000). This may contain dispersion contributions.

Index ranges: Limits on the Miller indices of the specified intensities.

**Table S2**: Details of hydrogen bond interaction geometry for **Sa•L-Arg•0.5H$_2$O**.			
Interaction	***d*** **(Å)**	***D*****(Å)**	***θ***
N1─H1A···O9	2.25(4)	3.017(4)	141(3)
N1─H1A···O10	2.43(4)	2.942(4)	115(3)
N1─H1B···O1	2.03(4)	2.898(4)	162(4)
N1─H1C···O7	1.99(4)	2.872(4)	159(4)
N2─H2A···O4	2.01(2)	2.843(4)	157(4)
N3─H3A···N10	2.24(4)	3.063(4)	153(3)
N3─H3B···O10	2.01(4)	2.892(4)	167(3)
N4─H4A···O6	2.14(3)	2.985(4)	156(4)
N4─H4B···O3	2.00(3)	2.878(4)	166(3)
N5─H5A···O8	1.90(3)	2.794(4)	170(3)
N5─H5B···O2	2.09(3)	2.820(4)	136(3)
N5─H5C···O4	1.95(4)	2.832(4)	167(4)
N6─H6···O6	2.09(2)	2.955(4)	167(4)
N7─H7A···O3	1.97(3)	2.856(4)	173(3)
N7─H7B···N9	2.15(2)	3.056(4)	171(3)
N8─H8A···O2	2.06(5)	2.836(4)	145(4)
N8─H8B···O1	2.02(4)	2.794(4)	144(4)
N9─H9C···O6	2.16(3)	2.948(4)	148(3)
N9─H9D···O10	2.12(4)	2.923(4)	150(4)
N10─H10C···O11	2.48(3)	3.195(5)	138(3)
N10─H10D···O11	1.93(4)	2.795(5)	162(5)
O11─H11C···O5	1.9400	2.770(4)	160.00
O11─H11D···O8	2.1000	2.921(4)	158.00
O11─H11D···O9	2.5900	3.005(4)	110.00
C4─H4C···O1	2.2800	3.242(5)	165.00
C5─H5D···N3	2.5200	2.890(5)	102.00
C10─H10A···O4	2.5300	3.455(5)	155.00

Figure S2: (**a**) Comparison showing the similarity of the calculated powder pattern and the experimental powder pattern of **Sa•L-Arg•0.5H_2O**, confirming a pure phase bulk synthesis; (**b**) The TG–DSC plot of **Sa•L-Arg•0.5H_2O**, revealing a broad endotherm corresponding to the loss of water molecule with an onset ≈ 55 ºC, followed by the decomposition of the anhydrous phase ≈ 150 ºC.

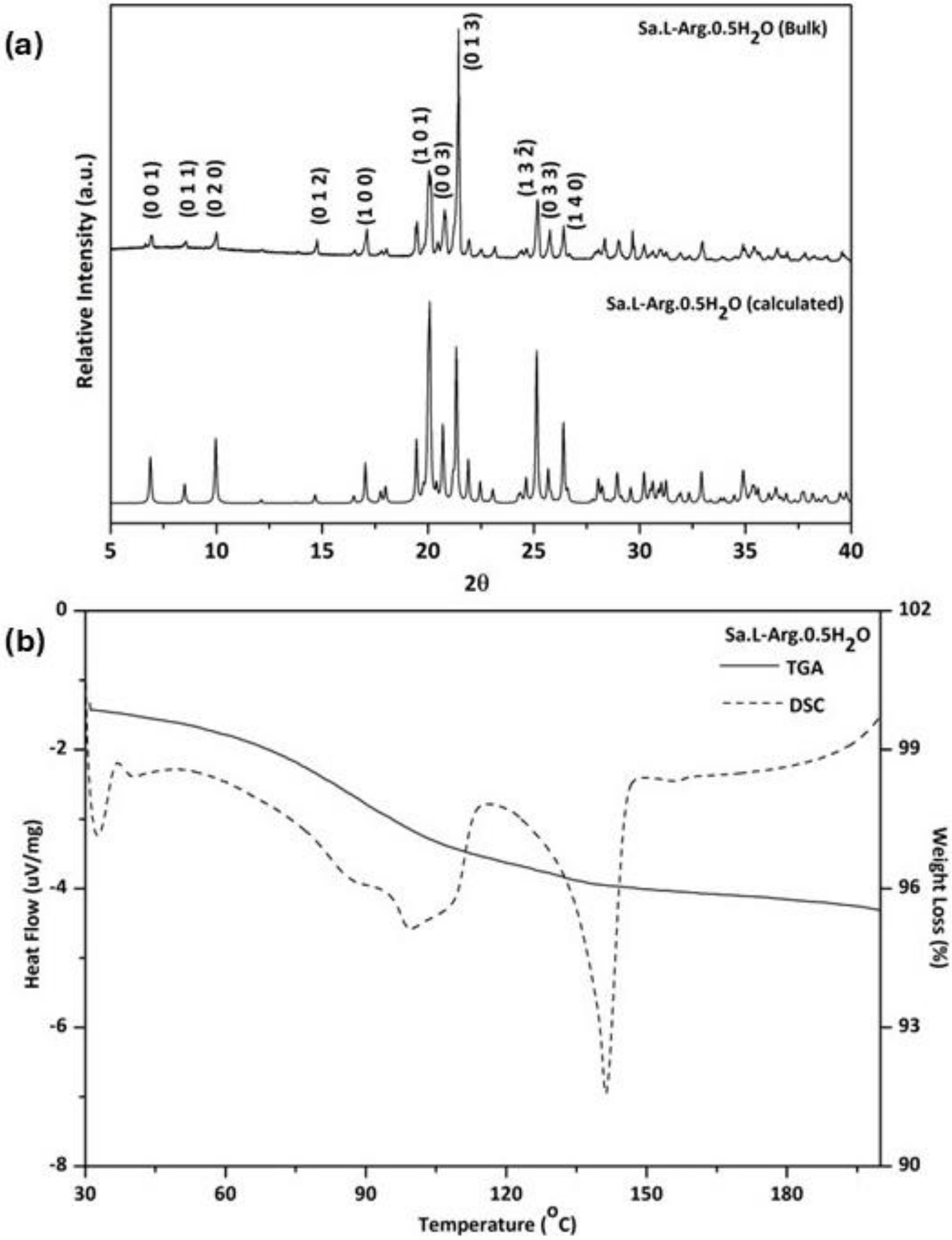

Figure S3: (**a**) Polycrystalline disc of **Sa•L-Arg•0.5H$_2$O** of diameter = 3.5 cm; (**b**) Average thickness of polycrystalline discs of **Sa•L-Arg•0.5H$_2$O** across five samples.

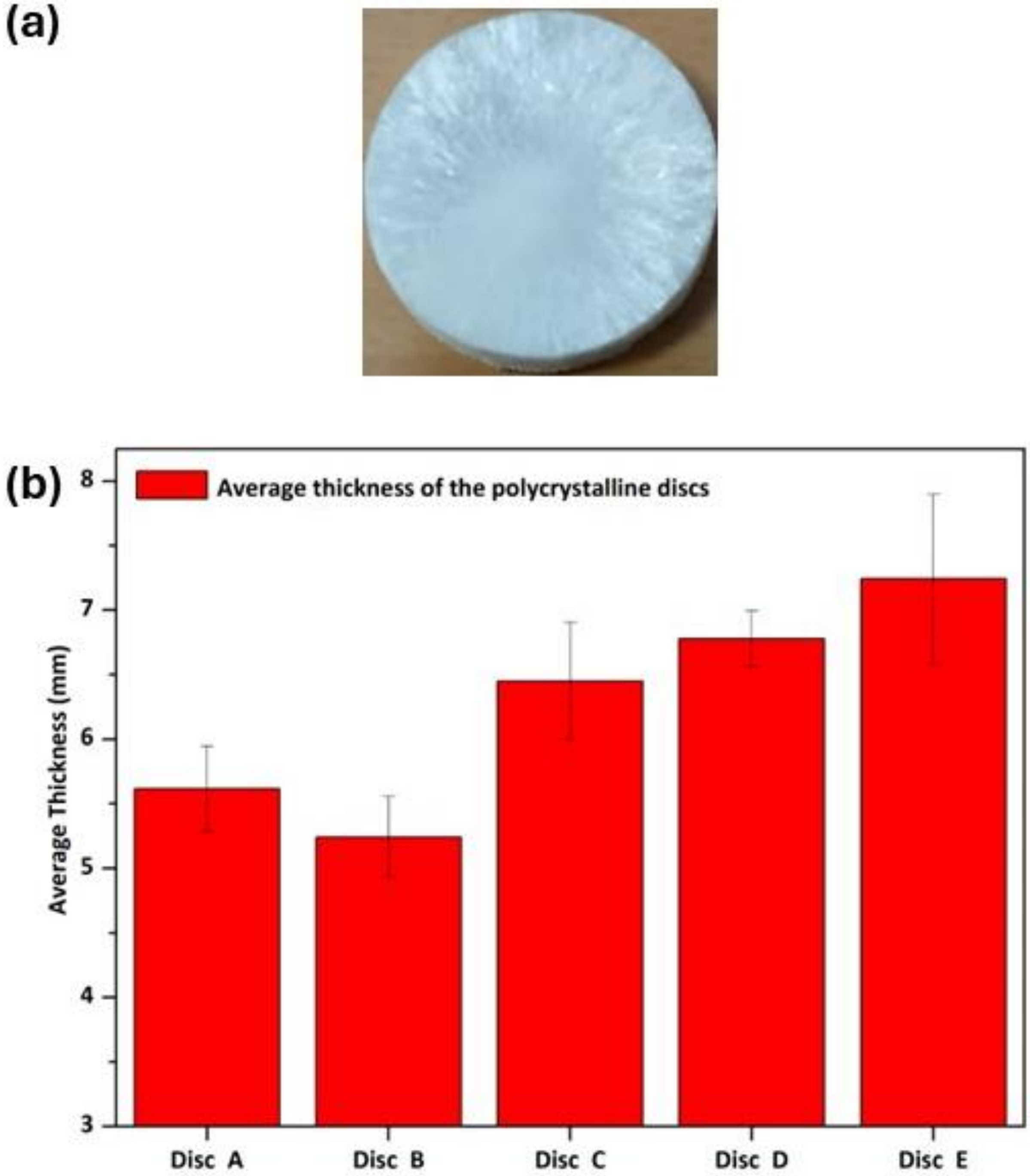

Figure S4: (**a**) and (**b**) SEM images of the surface and cross section of the polycrystalline disc of **Sa•L-Arg•0.5H$_2$O** showing condensed packing of aligned micro needle of **Sa•L-Arg•0.5H$_2$O**; (**c**) The elastic region of the Stress-Strain curve, from the initial linear region, of the circular discs obtained under a uniaxial force set to a maximum of 200 N.

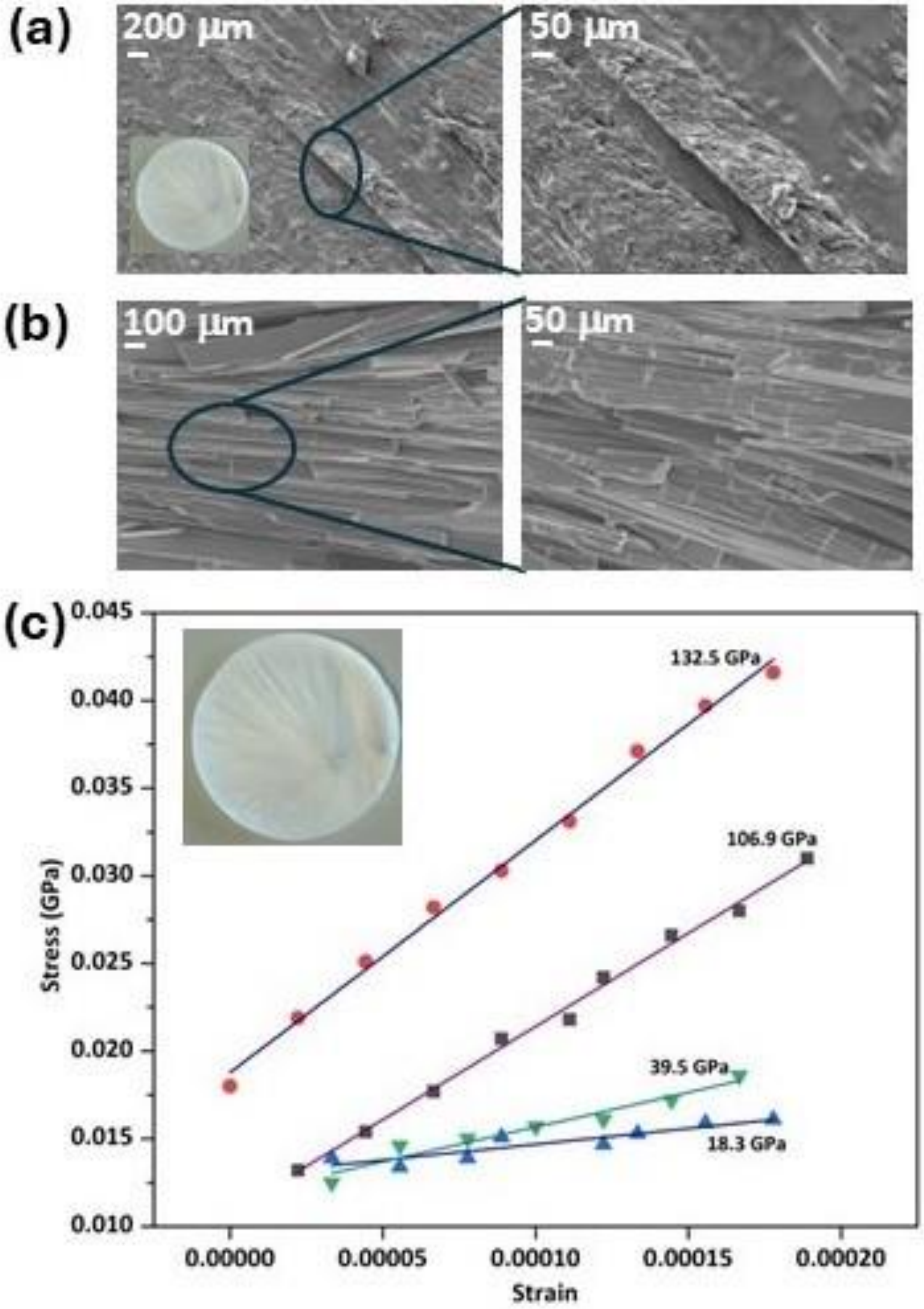

Figure S5: PFM topography, PFM Amplitude and PFM phase observed in nanoscale piezoresponse assessment of **Sa•L-Arg•0.5H_2O**.

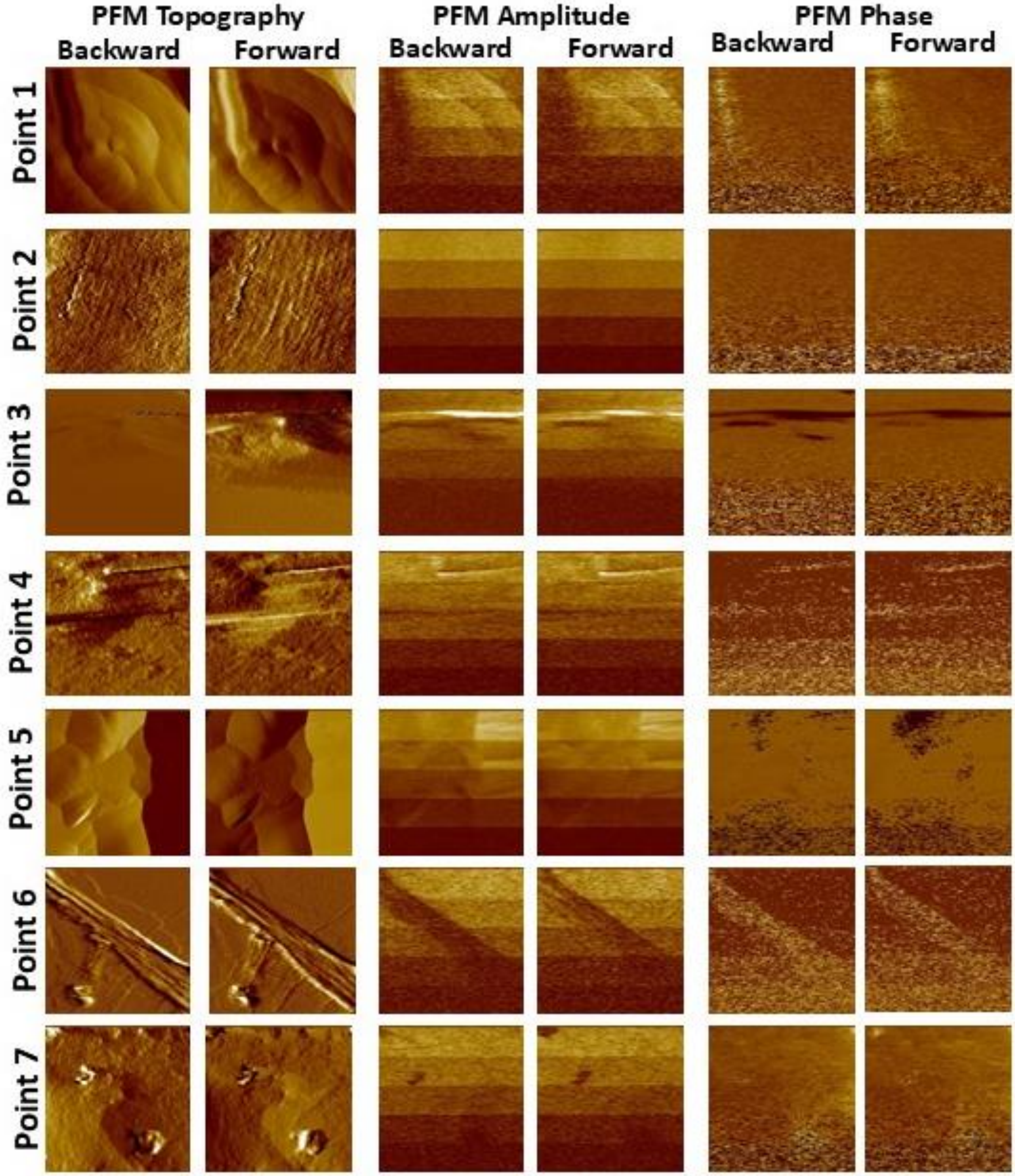

Figure S6: **a**) Schematic presentation of the steps followed for the Synthesis of **Ag@CC**; **b**) SEM images of (i) bare **CC** (ii) **Ag@CC** and (iii) magnified area for Ag nanoparticles on the **CC**; **c**) PXRD of bare CC and **Ag@CC**.

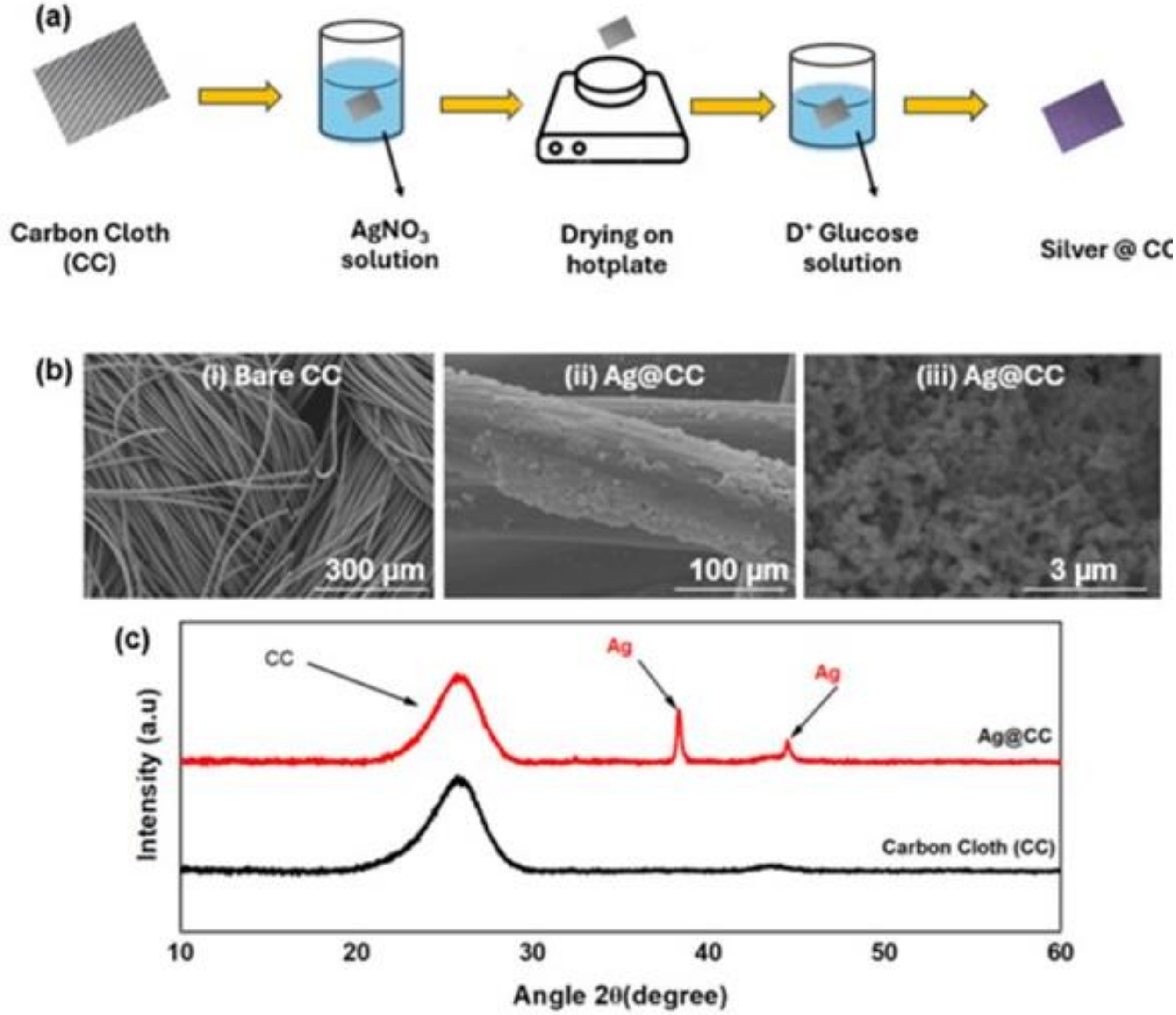

Figure S7 (**a**) SEM EDX results of **Ag@CC**; (**b**) Raman spectra of **Ag@CC**.

(a)

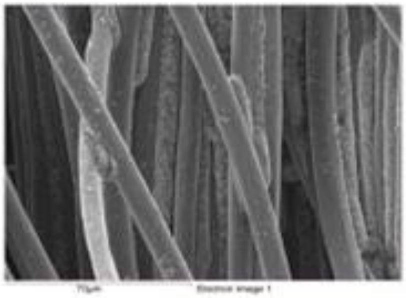

Element	Weight %	Atomic %
C K	60.03	85.50
O K	9.82	10.50
Ag L	19.26	3.06
Au M	10.89	0.95
Totals	100.00	

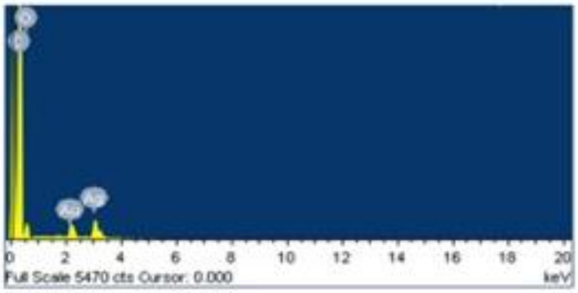

(b)

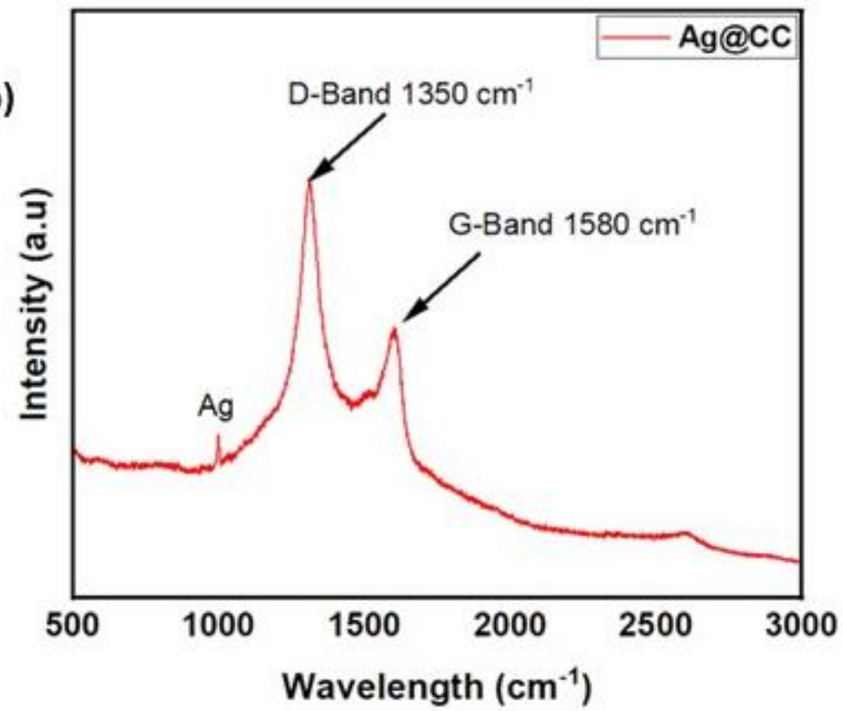

References:

1 G. M. Sheldrick, *Acta Cryst. A*, 2015, **71**, 3.

2 G. M. Sheldrick, *Acta Cryst. C*, 2015, **71**, 3.

3 O. V. Dolomanov, L. J. Bourhis, R. J. Gildea, J. A. K. Howard, and H. Puschmann, *J. Appl. Cryst.*, 2009, **42**, 339.

4 J. Hutter, M. Iannuzzi, F. Schiffmann, and J. VandeVondele, *WIREs Comput Mol Sci*, 2014, **4**, 15.

5 N. Argaman, and G. Makov, *Am. J. Phys.*, 2000, **68**, 69.

6 J. VandeVondele, and J. Hutter, *J. Chem. Phys.*, 2003, **118**, 4365.

7 J. VandeVondele, and J. Hutter, *J. Chem. Phys.*, 2007, **127**, 11410.

8 E. Matthias and E. S. Gustavo, *J. Chem. Phys.*, 1999, **110**, 5029.

9 J. P. Perdew, K. Burke, and M. Ernzerhof, *Phys. Rev. Lett.*, 1996, **77**, 3865.

10 G. Stefan, E. Stephan and G. Lars, *J. Comp. Chem.*, 2011, **32**, 1456.

11 G. Stefan, A. Jens, E. Stephan, and K. Helge, *J. Chem. Phys.*, 2010, **132**, 154104.

12 H. B. Richard, L. Peihuang, N. Jorge and Z. Ciyou, *SIAM J. Sci. Comput.*, 1995, **16**, 1190.
